# Data set of the toxic effects of divaricatic acid depside on *Biomphalaria glabrata* and *Schistosoma mansoni* cercariae^☆^

**DOI:** 10.1016/j.dib.2018.05.071

**Published:** 2018-05-19

**Authors:** H.A.M.F. Silva, W.N. Siqueira, J.L.F. Sá, L.R.S. Silva, M.C.B. Martins, A.L. Aires, F.F. Amâncio, E.C. Pereira, M.C.P.A. Albuquerque, A.M.M.A. Melo, N.H. Silva

**Affiliations:** a Laboratório de Produtos Naturais, Departamento de Bioquímica, Universidade Federal de Pernambuco, Recife, PE, Brazil; b Departamento de Energia Nuclear, Universidade Federal de Pernambuco, Recife, PE, Brazil; c Laboratório de Radiobiologia, Departamento de Biofísica e Radiobiologia, Universidade Federal de Pernambuco, Recife, PE, Brazil; d Laboratório de Imunopatologia Keizo Asami (LIKA), Universidade Federal de Pernambuco, Recife, PE, Brazil; e Laboratório de Geografia Ambiental, Departamento de Ciências Geográficas, Universidade Federal de Pernambuco, PE, Brazil

**Keywords:** Lichen substances, *Schistosoma mansoni*, *Biomphalaria glabrata*, Molluscicide activity

## Abstract

In this study, the molluscicidal and antiparasitic activities of divaricatic acid was evaluated, targeting the mollusc *Biomphalaria glabrata* and cercariae of the helminth *Schistosoma mansoni*. Divaricatic acid showed high toxicity against both adult snails (5.5 μg/mL) and embryos (20 μg/mL after 6 h of exposure). Similar activity was observed in *S. mansoni* cercariae after only a short exposure time. The divaricatic acid proved to be a promising substance for the control of the snail *B. glabrata*, an intermediate host of schistosomiasis, as well as the cercariae of the pathogen.

**Specifications Table**

**Table:** 

Subject area	*Chemistry, Biology*
More specific subject area	*Natural products biochemistry*
Type of data	*Table and figures*
How data was acquired	Stereoscopic microscope (Wild M3B, Heerbrugg, Switzerland)
Data format	Analyzed
Experimental factors	Divaricatic acid purification from *Ramalina aspera* lichen
Experimental features	Molluscicidal and embryotoxic activities on snails of the *Biomphalaria glabrata* species and the cercaricidal activity on *Schistosoma mansoni* of divaric acid were evaluated.
Data source location	*Recife, Brazil.*
Data accessibility	Data found in this article

**Value of the data**

• The data detail the embryotoxic, molluscicidal and cercaricidal activities of divaricatic acid, facilitating the correlation between the different tests and their concentrations, aiming to eliminate the vector in its different phases, and the etiologic agent of schistosomiasis in the same concentrations.
• The data provide a better understanding of the inviability/mortality information of *B. glabrata* used to obtain the lethal concentrations (LC_10_, LC_50_ and LC_90_) present in the original article.
• A more detailed view at the end of the analysis of the cercaricidal activity is provided by the expression of numerical data.

## 1 Date

**Fig. 1:**
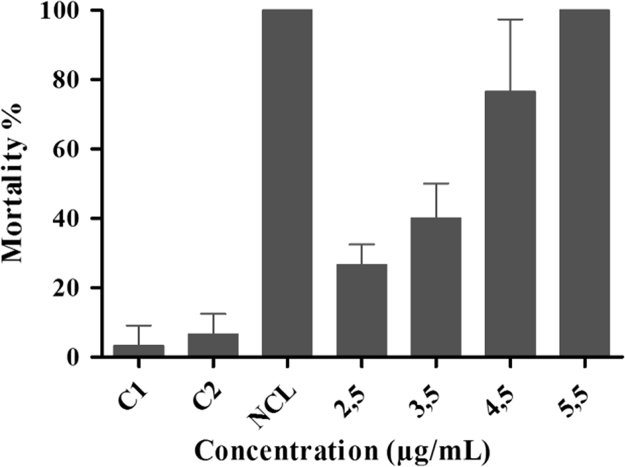
Mortality of *Biomphalaria glabrata* adult snails exposed to divaricatic acid. Control 1 (C1): filtered and dechlorinated water. Control 2 (C2): 0.5% DMSO in filtered and dechlorinated water. NCL: Niclosamide at a concentration of 1 µg/mL.

**Fig. 2:**
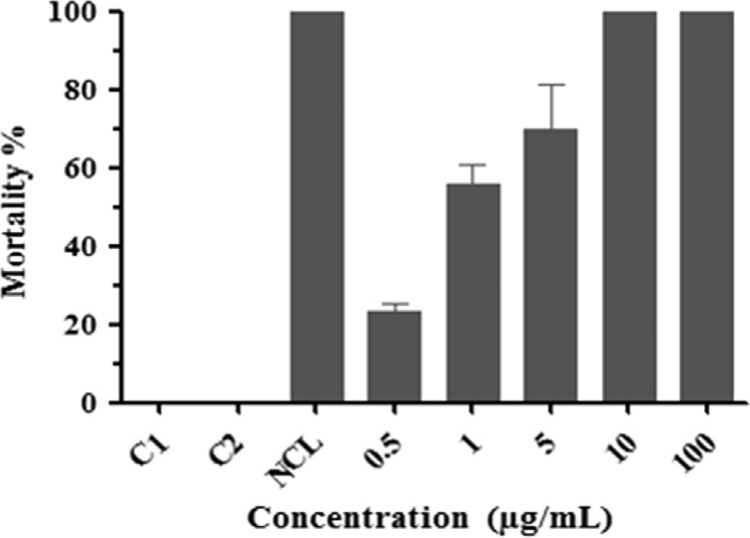
Mortality of *Schistosoma mansoni* cercariae exposed to divaricatic acid for 120 min. Control 1 (C1): filtered and dechlorinated water. Control 2 (C2): 0.5% DMSO in filtered and dechlorinated water. NCL: Niclosamide at a concentration of 1 µg/mL.

The data presented in this paper provide results related to embryotoxicity of divaricatic acid on *Biomphalaria glabrata* at different exposure times (6, 12, 18 and 24 h) (Table 1), as well as the molluscicidal activity of this compound on adult snails (Fig. 1) in 24 h of exposure. Data concerning the cercaricidal activity (*Schistosoma mansoni*) are shown in Fig. 2, where the percentage of dead organisms is reported at the final time of analysis (2 h of exposure to divaricatic acid).

**Table 1:** Inviability of *Biomphalaria glabrata* embryos subjected to divaricatic acid at different exposure times (6, 12, 18 and 24 h).

**Unviability by exposure period**				
**Experimental groups (µg/mL)**	**6 h ± SD**	**12 h ± SD**	**18 h ± SD**	**24 h ± SD**
**Control 1**	1.33 ± 1.15	1 ± 0.0	0.6 ± 0.57	1 ± 0.0
**Control 2**	3.33 ± 1.52	2.33 ± 0.57	3 ± 2.64	2 ± 1.7
**Niclosamide**	100	100	100	100
**Dicaricatic acid**				
***7.5***	0.6 ± 0.57	6.33 ± 1.52	6.33 ± 4.04	10.66 ± 7.3
***8.0***	1 ± 0.0	9.66 ± 4.61	19.66 ± 3.51	31.33 ± 19.0
***8.5***	1.66 ± 0.57	11 ± 1.0	32.66 ± 5.77	33.66 ± 28.5
***9.5***	1.66 ± 2.08	18 ± 6.92	35 ± 8.88	39.33 ± 8.0
***10***	8.33 ± 3.05	25.66 ± 8.38	39.66 ± 4.50	47.33 ± 16.2
***10.5***	10.33 ± 6.02	36.66 ± 11.68	49 ± 11.53	60.66 ± 9.0
***11***	15 ± 4.35	44.66 ± 9.01	55.33 ± 9.07	67.66 ± 17.1
***11.5***	19.33 ± 3.78	45.66 ± 4.93	67.33 ± 12.01	72.66 ± 6.8
***12***	25.66 ± 4.04	66.66 ± 12.42	75 ± 17.44	84 ± 15.3
***15***	81.66 ± 14.29	94 ± 5.19	96 ± 6.24	100
***20***	100	100	100	100

Control 1: filtered and dechlorinated water. Control 2: 0.5% DMSO in filtered and dechlorinated water. Niclosamide at a concentration of 1 µg/mL. Significant results were compared with control 2.

## 2 Materials and methods

### 2.1 Materials

#### 2.1.1 *Schistosoma mansoni* strain

BH strain, from Belo Horizonte, Minas Gerais, Brazil, maintained in Keizo Assami Immunology of the Federal University of Pernambuco (UFPE), through successive passages in snails of the species *Biomphalaria glabrata* kept in the Department of Tropical Medicine (UFPE).

#### 2.1.2 *Biomphalaria glabrata* molluscs

Geographical line from São Lourenço da Mata, Pernambuco, Brazil, maintained by successive generations in the Laboratory of Radiobiology of the Department of Biophysics and Radiobiology (UFPE).

#### 2.1.3 Divaricatic acid

Divaricatic acid was obtained from the ethereal extract of *Ramalina aspera* lichen and isolated according to the crystallization methodology of Asahina and Shibata [1] with modifications and its purity was confirmed by Thin Layer Chromatography [2] and High Performance Liquid Chromatography [3].

### 2.2 Methods

#### 2.2.1 Embryotoxicity test in B. glabrata

The assay was performed according to the methodology described by Oliveira-Filho and Paumgartten [4]. *B. glabrata* embryos in the blastula stage (*n* = 100) were exposed to divaricatic acid solubilized in 0.5% DMSO in different concentrations (7.5, 8.0, 8.5, 9.5, 10.0, 10.5, 11.0, 11.5, 12.0, 15.0 and 20 μg/mL), incubated for 6, 12, 18 and 24 h (25 °C ± 3) and subsequently washed with filtered and dechlorinated water (pH 7.0). The negative control was formed by two groups exposed to filtered and dechlorinated water (Control 1) and 0.5% DMSO solution (Control 2). Niclosamide (Bayluscide, Bayer) was used for the positive control [5], at a concentration of 1 μg/mL. Eight days after exposure, the embryos were analyzed for inviability (malformed embryos or dead) through a stereoscopic microscope and classified into embryos that were hatchlings and inviable (dead or malformed). The experiment was performed in triplicate.

#### 2.2.2 Lethality test in B. glabrata

The assay was performed according to the methodology described by World Health Organization [6]. Adults *B. glabrata* snails were exposed to concentrations of 2.5, 3.5, 4.5 and 5.5 μg/mL of divaricatic acid solubilized with 0.5% DMSO for 24 h (25 °C ± 3). The negative control was formed by two groups exposed to filtered and dechlorinated water (Control 1) and 0.5% DMSO solution (Control 2). Niclosamide (Bayluscide, Bayer) was used for the positive control [7], at a concentration of 1 μg/mL. The snails were observed daily and eight days after exposure, they were analyzed for lethality (absence of body movement, deep retraction into the shell, loss of hemolymph and absence of heartbeat).The test was performed in triplicate.

#### 2.2.3 Lethality test on Schistosoma mansoni cercariae

The assay was performed according to the methodology described by Santos et al. [8] with modifications. Snails of the species *B. glabrata* were exposed for 1 h in artificial light for the release of cercariae. For the test, approximately 100 cercariae were exposed to concentrations of 0.5, 1.0, 10.0 and 100 μg/mL of divaricatic acid. The divaricatic acid was solubilized in 0.5% DMSO. The negative control was formed by two groups exposed to filtered and dechlorinated water (Control 1) and 0.5% DMSO solution (Control 2). Niclosamide (Bayluscide, Bayer) was used for the positive control [9], at a concentration of 1 μg/mL. Afterwards, the cercariae were evaluated and counted for mortality after the 2 h period of exposure. The test was performed in triplicate.
