# Supplementary material for: Data set of the toxic effects of divaricatic acid depside on Biomphalaria glabrata and Schistosoma mansoni cercariae
Source: Data Brief. 2018 May 19;19:1393–7. doi: 10.1016/j.dib.2018.05.071 (PMC6139469; doi:10.1016/j.dib.2018.05.071)

**CONFLICT OF INTEREST STATEMENT**

The authors confirm that there are no known conflicts of interest associated with this manuscript, and there has been no significant financial support for this work that could have influenced its outcome.

In addition, we confirm that the manuscript has been read and approved by all authors and that there are no other persons who satisfied the criteria for authorship but are not listed.

We confirm that we have followed the regulations of our institutions concerning intellectual property, and those ones associated with this work and that there are no impediments to publication, including the timing of publication

The Corresponding Author is the sole contact for the Editorial process (including Editorial Manager and direct communications with the office), and responsible for communicating with the other authors about progress, submissions of revisions and final approval of proofs.

All authors confirm that have provided a current, correct email address which is accessible by the Corresponding Author and which has been configured to accept email from williams.wns@gmail.com.

Signed by all authors as follows:


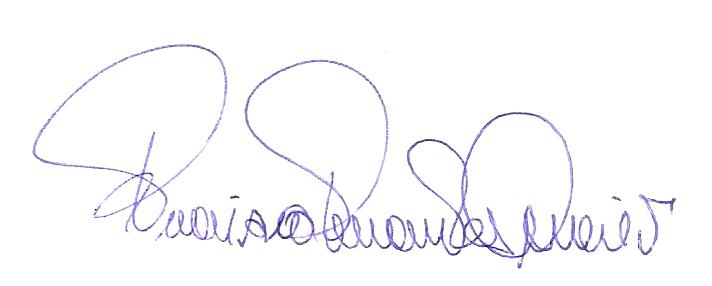

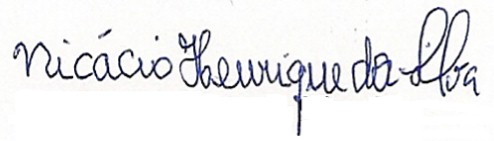

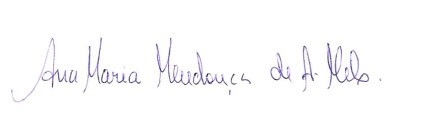

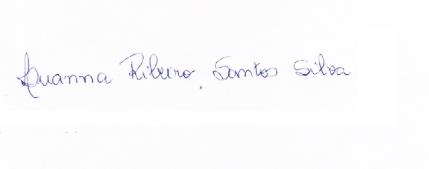

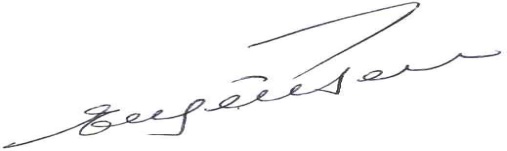

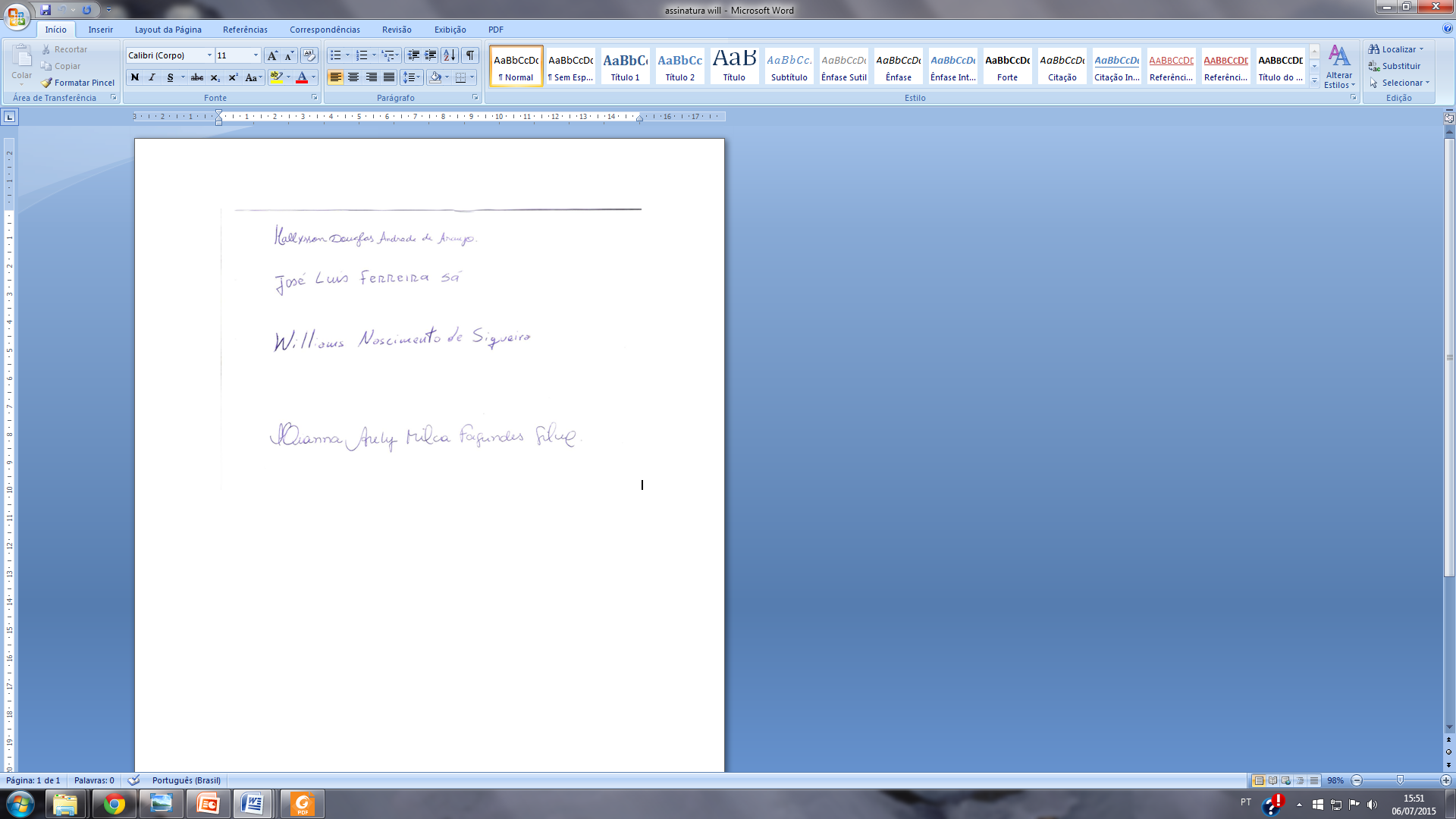

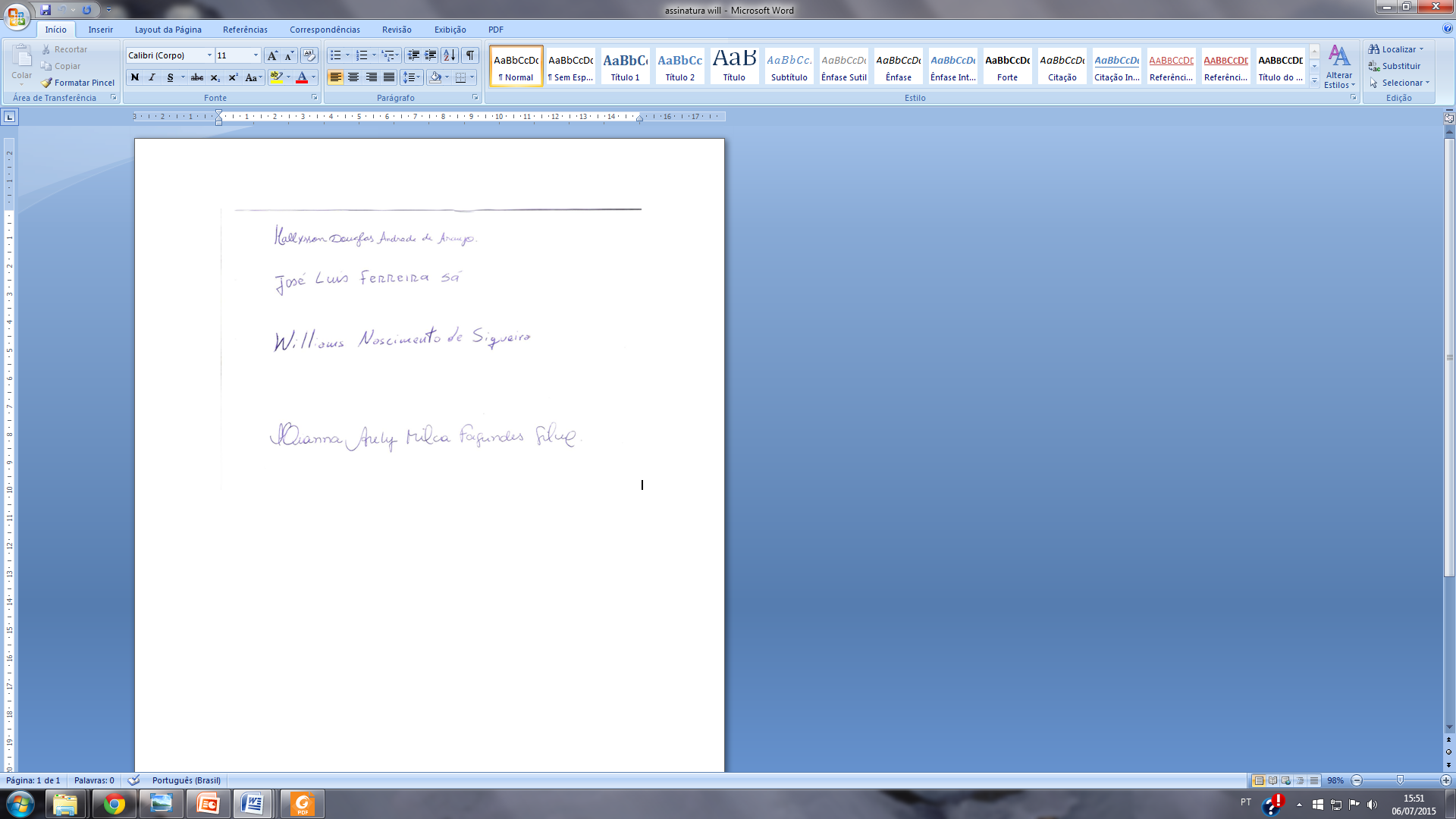

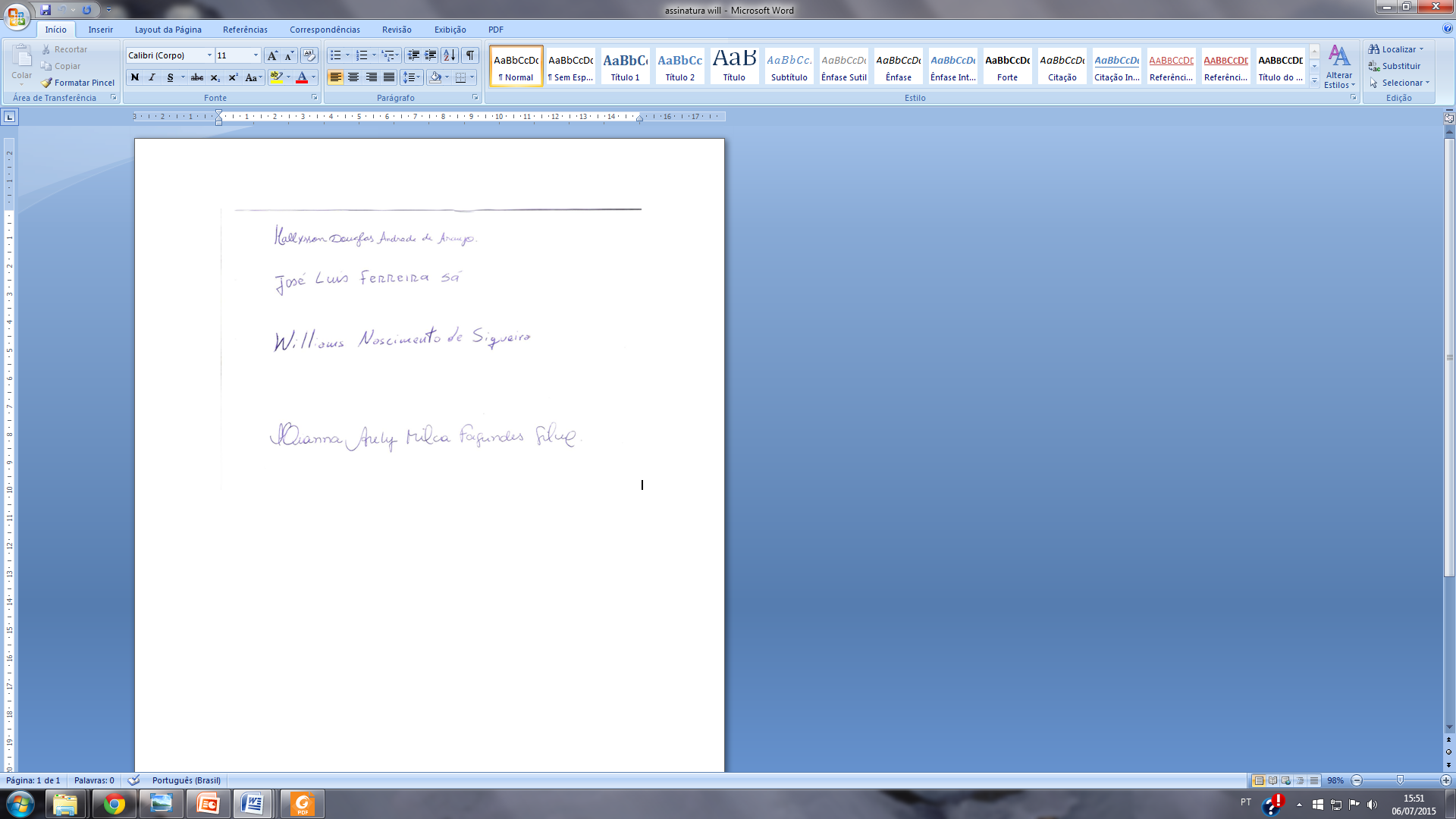

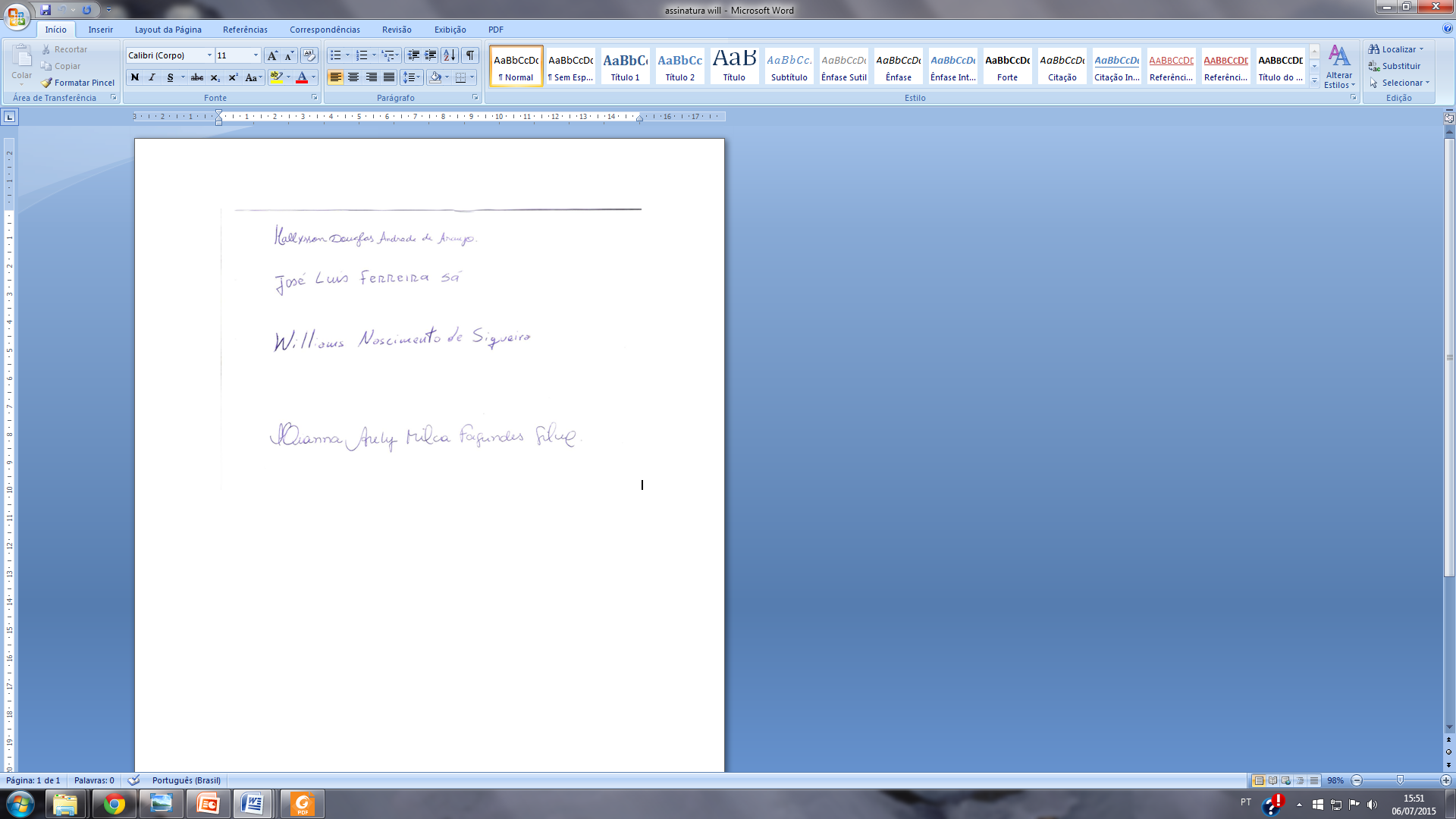

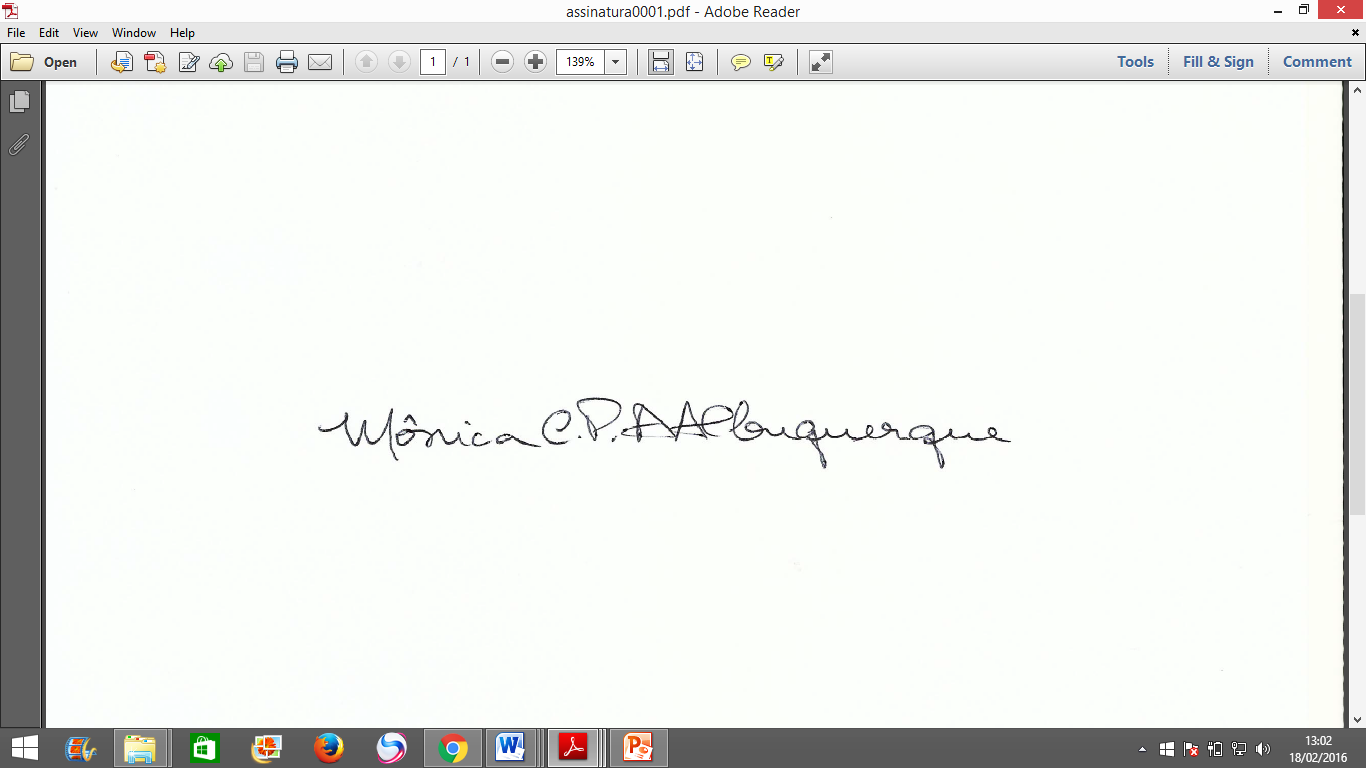

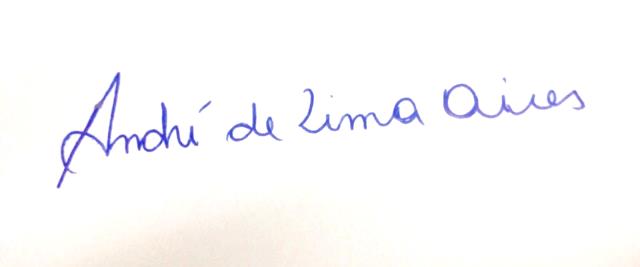

Supplement: Supplementary file 1 — Supplementary material [file mmc1.doc]
